# The *Croatian Medical Journal* – evidence for better health

**DOI:** 10.3325/cmj.20026.67.253

**Published:** 2026-08

**Authors:** Mario Šekerija

**Affiliations:** *Croatian Medical Journal*, Zagreb, Croatia

When I began my term as the editor-in-chief of the *Croatian Medical Journal* (*CMJ*), many things crossed my mind. Among the more immediate ones were not only how a medical journal should be managed but also what a medical journal today should be.

For my generation of medical students in Zagreb, the *CMJ* was *the* medical journal. This experience was partially shaped by the second-year course Introduction to Scientific Work in Medicine taught by the then editor-in-chief, Matko Marušić. There, we adopted the idea that scientific thinking and evidence are the foundations of modern medicine and that they can directly improve our future professional skills. This thought has been with me ever since, and today I am especially glad that I can pass it on to new generations. The first manuscript on which I was the corresponding author was initially submitted to the *CMJ*, and promptly received a very constructive desk rejection. This experience I apparently share with at least one former *CMJ* editor-in-chief (1).

The *CMJ* has always had a distinctive role in connecting Croatian (and regional) science with the international scientific community. The combination of author-helpful editorial policy (2), insistence on research integrity, and openness to various fields of medicine has made it a key publication outlet for biomedical science in Croatia. This has never been only about indexing in international databases, the impact factor, and increasing citation counts. It has also been about generations of young doctors and scientists growing up with the idea that if they study hard and apply what they were taught in medical school, they too will be able to publish in such a journal.

Every new editor-in-chief offers a different outlook on the journal. I come from epidemiology and public health, where we were trained to think about determinants of health and disease in different populations, possibilities of disease prevention and health promotion, assessment of health inequalities, and the organization of health systems. I believe this can bring a fresh perspective to outlining the purpose of a general medical journal beyond publishing science-based findings: asking how these findings can contribute to the populations we serve.

In my daily work as the head of the Croatian National Cancer Registry, I have noticed a parallel between cancer registries and scientific journals. Population-based cancer registries collect data on malignant diseases in a geographically defined population. These data are then classified, validated, interpreted, and disseminated to provide reliable information on cancer (3). Scientific journals do similar work: they subject manuscripts to editorial assessment and peer review to make them clearer, more rigorous, and more useful contributions to scientific knowledge. Neither registries nor scientific medical journals should only be repositories of data or scientific papers. Their full value is visible when the information they help to produce leads to a better understanding of disease and contributes to improved prevention, health care, public policy, and further research. Additionally, the future of both cancer registries and scientific journals is already being affected by new technologies (3,4).

All these issues are especially relevant at a time of abundant and interconnected health data, and the increasing volume of scientific publishing and editorial burden. The new frameworks for the responsible secondary use of health data (5) offer substantial opportunities for evidence-based research and policymaking, but they also raise a number of questions about quality, interoperability, privacy, ethics, and public trust, as well as the repetitive and methodologically uncritical use of publicly available data sets. Also, the expanding use of artificial intelligence tools adds another layer of capabilities and responsibilities. These tools can do a lot to improve research and editorial workflows, but their use should remain transparent, accountable, and consistent with the evolving standards for responsible scientific publishing (4). For the *CMJ*, these circumstances mean that we have to maintain rigorous methodological and ethical principles while making editorial decisions as clear, timely, and constructive as possible.

Our goal is to remain open to various disciplines and methods, continue to support research from smaller scientific communities, and work on transparent policies for the responsible use of data and artificial intelligence in medicine. We invite our authors, reviewers, and future collaborators to help us shape this next chapter in the life of the *CMJ*.

I take on this role with deep respect for the history of the *CMJ* and the previous editors-in-chief (Matko Marušić, Ana Marušić, Ivan Damjanov, Srećko Gajović, Tomislav Smoljanović, and Svjetlana Kalanj Bognar), with sincere gratitude for the warm welcome by the editorial team, and with responsibility to our readers and the wider community around the journal.

As my predecessor stated in her outgoing editorial, “The *CMJ* sails on, with confidence and caution” (6). Our founding editors urged their successors to leave the safe harbor and “Explore! Dream! Discover!” (7). While this suggestion applies well to the journal management, I believe that a safe harbor is sometimes what early-career authors need and what makes their voyage possible. I will work on the *CMJ* so that it continues to be that place for authors: a place where ideas can be tested, manuscripts strengthened, and researchers encouraged to go boldly into wider scientific seas. The winds will certainly continue to change, but our course should remain steady and the mission clear: to help reliable evidence become useful knowledge, so that this useful knowledge can contribute to better health.
